# Health literacy – engaging the community in the co-creation of meaningful health navigation services: a study protocol

**DOI:** 10.1186/s12913-018-3315-3

**Published:** 2018-06-28

**Authors:** Christine Loignon, Sophie Dupéré, Martin Fortin, Vivian R. Ramsden, Karoline Truchon

**Affiliations:** 1 Faculty of Medicine and Health Sciences, Department of Family and Emergency Medicine, Université de Sherbrooke, Québec, Canada; 2 Faculty of Nursing, Université Laval, Québec, Canada; 3 Department of Academic Family Medicine, College of Medicine, University of Saskatchewan, Saskatchewan, Canada

**Keywords:** Health literacy, Participatory action research, Knowledge translation, Co-creation, Health literacy, Aboriginal peoples, Underserved populations

## Abstract

**Background:**

A large proportion of patients encounter barriers to access and navigation in complex healthcare systems. They are unable to obtain information and services and to take appropriate action to improve their health. Low health literacy affects the ability of individuals to benefit from health services. Some social groups are disproportionately affected by low health literacy, including those with low educational attainment, Aboriginal people, and those on social assistance. These individuals face significant barriers in self-management of chronic diseases and in navigating the healthcare system. For these people, living in a context of deprivation contributes to maintaining disparities in access to healthcare and services. The objective of this study is to support knowledge co-construction and knowledge translation in primary care and services by involving underserved and Aboriginal people in research.

**Methods:**

This study will integrate participatory health processes and action research to co-create, with patients, individuals, and community members impacted by health literacy, practical recommendations or solutions for facilitating navigation of the healthcare system by patients, individuals, and community members with less than optimal health literacy on how to best access health services. With this approach, academics and those for whom the research is intended will collaborate closely in all stages of the research to identify findings of immediate benefit to those impacted by health literacy and work together on knowledge translation. This study, carried out by researchers, community organizations and groups of people with low health literacy from three different regions of Quebec and Saskatchewan who can play an expert role in improving health services, will be conducted in three phases: 1) data collection; 2) data analysis and interpretation; and, 3) knowledge translation.

**Discussion:**

Persons with low health literacy experience major obstacles in navigating the health system. This project will therefore contribute to addressing the gap between healthcare challenges and the needs of underserved patients with multi-morbidity and/or low health literacy who have complex health-related needs. It will pave the way for co-creating successful solutions for and with these communities that will increase their access to health services.

## Background

Health systems represent a large burden on public finances in Canada and in many industrialized countries. They should contribute more to reducing social inequalities in health [1]. However, there is a body of research pointing to the lack of recognition of the social causes of health inequalities in the healthcare system in Canada and elsewhere [2–7]. Likewise, literacy, which plays a central role in social inequalities in health, is given little consideration within health systems. Yet, literacy is now recognized as an important determinant of health that is closely linked to other social determinants of health in Canada [8]. Low literacy is associated with low health literacy. Health literacy (HL) refers to a set of skills and abilities for finding, understanding, evaluating and, communicating information in a way that promotes and improves health [9–11].

In Canada, nearly 60% of adults are unable to obtain health information and services, to understand and take appropriate action to improve their health status, and to make the appropriate decisions for themselves to improve their condition [12]. Some social groups are disproportionately affected by a low level of HL, including: people with low education, Aboriginal people, and those on social assistance [7, 13–17]. These individuals face significant barriers in self-management of chronic diseases and in navigating the health system. The context of deprivation and social vulnerability in which they live contributes to maintaining disparities in access to health care and services [18]. Some studies point to the need to adapt services to individuals’ HL by engaging these same people in the co-production of health services [19–24].

### Literacy and research on chronic diseases

Low literacy levels have a significant negative impact on people’s health and quality of life, as well as on the quality and cost of healthcare [25, 26]. According to Mitic and Rootman, “One of the ways to address the anticipated escalation in chronic disease rates and the subsequent demands this will place on the health care system is to engage patients in more effective self-management.” [26].

In fact, the populations most likely to have difficulties with self-management are those with low literacy levels [15, 27]. Intervention programs for prevention or support for chronic disease self-management are not sufficiently responsive to the needs of these individuals [19, 26]. They face several individual and systemic barriers to navigating the healthcare system, have less access to prevention and screening programs and preventive care that lead them to emergency consultation or to experiencing a deterioration in their health status, an issue of concern that challenges the efficiency of the health system [28]. It is very important to address these barriers preventing access to healthcare and care that is adapted to people’s low literacy skills, as they are preventable [29–34].

To our knowledge, very little research has focused on working with people with low literacy skills to identify solutions for overcoming individual and systemic barriers to navigating health care system [20]. Participatory research could be a relevant avenue: it requires time and sustained investment by all partners, but it is recognized for generating solutions that are relevant and adapted to people’s reality [3, 35].

### Involvement of patients with low literacy levels in research

As part of this participatory research project, we want to actively involve people with low health literacy who have encountered barriers to self-management and health system navigation. The definition of people with low health literacy is that they have a low “ability to access, understand, evaluate and communicate information as a way to promote, maintain and improve health in a variety of settings across the life-course” [15]. Decision-makers and clinicians involved in our work find it difficult to reach these people. Among other things, they expressed a wish to involve these people in the design of chronic disease prevention tools that are adapted to individual social capacities and cultural values.

The added value of patient engagement in health services research is increasingly recognized and valued through initiatives such as INVOLVE, PCORI, and the CIHR Framework for Engagement [36–38]. However, we do not have established knowledge of the factors that facilitate this engagement for people with low health literacy. Moreover, there is very little literature on how best to involve people with low literacy skills in research on the development of appropriate healthcare tools and programs for chronic diseases.

Involving people with low research literacy is crucial because they are often excluded from health services, making them invisible, especially in research based on medical administrative databases. Moreover, they are also less well represented in surveys, partly due to the difficulty in reaching them and encouraging them to participate in research, or to attrition [39]. We recently conducted a literature review on the participation of vulnerable people in participatory primary care research that identified important benefits but also significant shortcomings [40]. In particular, we identified that vulnerable people have been instrumentalized in several research projects, mostly passively participating in data collection and analysis. There are therefore significant challenges to engaging patients with low literacy skills in research, which points to the relevance of collaborating with experts in community work.

The engagement of patients/individuals with low health literacy is crucial to the research because these people 1) are more likely to be living with multiple chronic conditions without easy access to healthcare services; and 2) are under-represented in primary care innovation-focused research.

Underserved patients/individuals and communities frequently do not have a family physician and as a result have less than optimal experiences with the health system, despite having complex needs in terms of healthcare and services [32]. Even if they have a family physician, they may end up in at emergency services due to a deterioration of their health and well-being leading to hospitalization. According to the data generated by several studies, including EQUihealThY [3], patients encounter significant barriers in navigating the health system and often feel negatively judged by their physicians. Some studies have observed that physicians feel poorly equipped to meet the needs of patients with complex needs that exceed their medical skills [41].

### Research objectives

The goal of this project is to support knowledge co-construction and translation with respect to front-line care and services, by engaging underserved or Aboriginal people with low literacy skills in the research. The specific objectives are as follows:

1. Identify key challenges related to self-management and navigation of the health system by identifying convergences and discrepancies among three differentiated groups of patients with low health literacy;
2. With each group of patients with low health literacy, identify practical solutions (for clinicians, policy-makers, and researchers) to address a key challenge;
3. Document, based on patient experience, the conditions for active participation in research and the relevant modalities (tools, procedures and practices) in a low-literacy setting.

## Methods/design

This project is based on a participatory research design combined with a qualitative approach [42–44]. The participatory approach we use favours active and equitable participation of non-academic researchers in all stages of research, from the research question to the dissemination of results [45–47]. These non-academic researchers (patients and leaders of community organizations) can thus derive immediate benefits from the research and be involved in knowledge translation [45].

This project is based on participatory health processes integrated with action research that engages patients, individuals, and the community in the process of co-creating meaningful services and programs. Such an approach is optimal for engaging these participants in co-creating solutions and practice innovations that will facilitate improved patient-centred care and self-care. With this approach, both academics and those for whom the research is intended will collaborate closely in all stages of the research to achieve immediate benefits for those affected by health literacy, and work together on all aspects of knowledge translation.

This project thus aims to lay the groundwork for a long-term partnership between researchers, community organizations, and groups of people with low health literacy from three different regions of Quebec and an Aboriginal community in Saskatchewan. Non-academic researchers from the community are considered experts for the purpose of improving health navigation services. This project will involve two participatory health research teams: one in Quebec and one in Saskatchewan. Each province’s research team will comprise both academic and non-academic researchers/peer researchers with whom a strong partnership or relationship already exists. We will ensure joint meetings of the research teams to facilitate the sharing of data among the knowledge users/peer researchers and between provinces. Loignon, Ramsden, and at least two patients or community members per group will participate in regular Steering Committee meetings. This Inter-Jurisdictional Steering Committee will monitor research progress and be responsible for ensuring sustainability. Using participatory health processes integrated with action research, the goal is to co-create innovative solutions that aim to improve health literacy and navigation of the health system by underserved patients/individuals and/or communities. In this project, we hope to pursue and expand this collaboration with patients/individuals in the communities through an interprovincial partnership.

### Participants and sampling

In Quebec, our project includes three groups of patients from the community, some of whom are members of community organizations: one in Montreal (Group 1), a second in Chicoutimi (Group 2), and the third in Québec City (Group 3). These three urban settings are different in terms of socio-cultural characteristics, but all include vulnerable clienteles with different literacy challenges. Group 1 includes illiterate people and immigrants who have difficulty expressing themselves in French or English, Group 2 is composed of multi-morbid people (more than three chronic diseases), and Group 3 includes illiterate people. The activities of Group 2 are financially supported by the Fondation de ma Vie, a private foundation. In Saskatchewan, Ramsden has set up a group (Group 4) through contacts in the Aboriginal community in Saskatoon.

Patient selection is based on convenience sampling. Mixed strategies are used for sampling because the target patients or community members are usually difficult to recruit as they may have distrust of researchers. In participatory research, the aim is not to aspire to representative sampling, because generalization is theoretical and the study must be transferable to another context. Morreover, according to a recent systematic review of the literature, there is no comparative research to demonstrate the effectiveness of a particular selection method in patient engagement projects [38]. In this project, we are experimenting with two methods of patient selection: through community contacts (Groups 1,3, and 4) and through links to the clinical setting (Group 2).

In addition, although we will adopt the same approach, group accompaniment will necessarily vary across groups in some respects (e.g., facilitation style, size, key themes, etc.). This diversity of experience is a strength that will fuel our reflection on the conditions conducive to the participation of people in research projects. The patients selected live with low health literacy and encounter barriers to self-management and to navigating the healthcare needed for their chronic diseases. These criteria will be validated during recruitment through the expertise of community organization workers or community organizers. Each group will include a maximum of 20 patients, ensuring diversity in terms of age, gender, occupation, cultural background, and level of research experience. This number is set high to compensate for attrition and is based on our previous experience with patients. By paying particular attention to people’s living conditions and supporting them, for example by avoiding meetings at the end of the month, which are often more difficult for people on low income [48], we expect about ten patients to participate on average in each meeting.

This research will be conducted in three participatory phases: 1) data collection; 2) data analysis and interpretation; 3) knowledge translation.

### Phase 1: Data collection

#### Concurrent meetings of each group

We anticipate that each group will need to meet on a regular basis for at least one monthly 2- to 3-h meeting. The first two meetings will be used to take ownership of the project and adjust the work plan as necessary. This will provide an opportunity to clarify the roles and responsibilities of each group member. It will also allow each group member some time to speak and to have an initial exchange on the difficulties and challenges faced by patients. The content of the meetings will focus on solutions that can be put in place to address the challenges faced by patients with low health literacy.

These meetings will be co-facilitated by a researcher and a community organization leader or community health professional who is familiar with the challenges faced by people with low literacy skills. These people also have expertise in facilitating groups of people in a way that supports mutual trust and respect. They will be paid according to the salaries of their home organizations. Patients will be compensated for the time spent in the meetings. Researchers will play a facilitating role and attend group meetings as needed.

In addition, for two of the groups in Quebec, members will use digital storytelling as a tool to collect and disseminate data [49, 50]. The choice to use this research method was made by our community partners and members of these groups. Digital storytelling allows people who are unused to expressing themselves in public speak their mind on a given topic – in this case access barriers related to health literacy and navigation, and solutions for improving health – by making a short autobiographical film of 60 to 210 s narrated in the first person. The film works resulting from this methodological approach use visual and audio materials that the directors make specifically to illustrate their subject matter or that they select from their personal archives [51].

### Phase 2: Data analysis and interpretation

#### Pooling the work and expertise developed in the groups

We plan to describe and analyze our work within the groups at each site comparatively. To do this, we will prepare fact sheets with the help of research assistants summarizing the knowledge acquired in each group during Phase 2, which will be shared with all members. We will then work more closely with five people in each site (1 researcher, 1 practitioner, and 3 patients) who will be the spokespersons for their group. We are planning two 2-h videoconference meetings (Webex) between the groups to discuss the various experiences, modalities, and impacts observed and anticipated after the meetings. We will prepare a summary report of this sharing process, which include an analysis of the experiences, followed by a summary description of the work carried out by the groups as well as the facilitating modalities that will have enabled authentic participation of the people involved.

### Phase 3: Knowledge translation

We will involve patients who wish to do so in the dissemination activities. Our experience in this area has shown that these activities benefit patients, who feel valued, as well as the various audiences that have access to lived expertise that supports the relevance of the research. We will involve patients in creating video capsules about their experience. These capsules will be used in training activities for professionals, managers, and decision-makers. We will also train patients to present them to various audiences (clinicians, decision-makers, and researchers) using the expertise of Dupéré and Loignon in this area.

The ideas that evolved from engaging with patients/individuals in exploring what would improve their visits with a health care provider have led to one co-creation, namely, the development of a wallet card. We will also build an online app so that patients/individuals can better navigate the healthcare system and more optimally engage in self-care.

### Ethical considerations

Each participant will sign a consent form or provide oral consent before participating in the study. This study protocol, including recruitment procedures, was approved by the Centre Intégré Universitaire de Santé et des Services Sociaux (CIUSSS) de l’Estrie and Chicoutimi. The project has also been approved by the Ethics and Research Committee of Université de Laval. The project in Saskatchewan was submitted to the University of Saskatchewan’s Behavioural Research Ethics Board Programs and was deemed to be Exempt from Ethical Review.

## Discussion

This project will contribute to addressing the knowledge gap concerning healthcare challenges and the needs of underserved patients/individuals with multi-morbidity and less than optimal health literacy who have complex health-related needs. It will pave the way for successful solutions to be co-created with these communities that will enhance health literacy and increase their access to health services.

Persons with less than optimal health literacy, including Aboriginal peoples, persons living in poverty, and non-English or French speaking new Canadians, experience obstacles in navigating the health care system in every province in Canada, including Quebec and Saskatchewan. In Quebec, especially in Montreal, a city where these populations have major needs in terms of healthcare, we lack data on which innovations might well be adapted to their complex needs. In Saskatchewan, improving the capacity of the healthcare system to meet the complex needs of Aboriginal and underserved communities is of high importance. For example, 53% of the Quebec population does not have a literacy level to function optimally. And 19% of the Quebec population aged 16 to 65 (i.e. 1 in 5) have great difficulty in reading and writing [52, 53]. A bottom-up approach to health literacy which evolved from the Patient’s Medical Home [54] is expected to enhance patient/individual visits and improve physician/nurse practitioner communication while at the same time assisting patients/individuals with navigating the health system.

This project team has expertise in participatory research and patient-engaged research. We are also involved in various projects aimed at strengthening innovations in primary care for persons who have complex needs in terms of health and health care (PaCE and IMPACT). This project will generate knowledge about the challenges encountered by patients with multi-morbidity and low health literacy who have complex needs. It will pave the way for co-creating with these communities successful solutions that will increase their access to health services. The solutions identified will be co-created with the knowledge and experience from peer researchers (community members), and the scientific evidence will be transferable to other similar contexts, including urban, rural and remote communities in industrialized countries with universal health systems. Thus, we plan to share the results, findings, and tools that evolve from the project through the engagement of patients, individuals, and communities.

## Abbreviations

CIHR: Canadian Institutes of Health Research
EQUIhealThY: Abbreviated name of a participatory research we conducted, https://www.ncbi.nlm.nih.gov/pmc/articles/PMC4300157/)
FQRS: Fonds de recherche en santé du Québec
HL: Health literacy
IMPACT: Name of the research program lead by J Haggerty.
INVOLVE: Organization funded by National Institute for Health Research in United Kingdom to support public participation in research, http://www.invo.org.uk/
PACE: Name of the research program lead by M Stewart and M Fortin.
PCORI: Patient Centered Outcomes Research Institute
